# The Roles of Oxidative Stress and Red Blood Cells in the Pathology of the Varicose Vein

**DOI:** 10.3390/ijms252413400

**Published:** 2024-12-13

**Authors:** Lukasz Gwozdzinski, Anna Pieniazek, Krzysztof Gwozdzinski

**Affiliations:** 1 Department of Pharmacology and Toxicology, Medical University of Lodz, 90-752 Lodz, Poland; 2 Department of Oncobiology and Epigenetics, Faculty of Biology and Environmental Protection, University of Lodz, 90-236 Lodz, Poland; anna.pieniazek@biol.uni.lodz.pl (A.P.); krzysztof.gwozdzinski@biol.uni.lodz.pl (K.G.)

**Keywords:** varicose vein, oxidative stress, red blood cell, reactive oxygen species

## Abstract

This review discusses sources of reactive oxygen species, enzymatic antioxidant systems, and low molecular weight antioxidants. We present the pathology of varicose veins (VVs), including factors such as hypoxia, inflammation, dysfunctional endothelial cells, risk factors in varicose veins, the role of RBCs in venous thrombus formation, the influence of reactive oxygen species (ROS) and RBCs on VV pathology, and the role of hemoglobin in the damage of particles and macromolecules in VVs. This review discusses the production of ROS, enzymatic and nonenzymatic antioxidants, the pathogenesis of varicose veins as a pathology based on hypoxia, inflammation, and oxidative stress, as well as the participation of red blood cells in the pathology of varicose veins.

## 1. Introduction

Despite many studies, the etiology and pathogenesis of primary varicose veins have not been fully elucidated. Chronic cardiovascular disease (CVD) denotes a disease of the cardiovascular system, while chronic venous insufficiency (CVI) is a broader term that includes venous disease that occurs when the veins in the legs are damaged [1]. It is postulated that the main cause of the formation and development of varicose veins is blood stasis in the vessel caused by damaged valves. The consequence of this condition is an increase in venous pressure and venous reflux, which contributes to the development of the disease. Oxygenation of the vein occurs by the diffusion of oxygen from the blood flowing through the vessel and from the vasa vasorum. Although there is no direct evidence of differences of the oxygen content in the damaged vessel and in the normal vessel, hypoxia-inducible factors (HIFs) are activated in response to hypoxia [2,3]. HIFs regulate the expression of many downstream genes involved in many processes, such as cell metabolism, cell proliferation, immune response, infection by pathogens, cell growth/death, and many others. Studies conducted on cell cultures have shown that hypoxia caused activation of endothelial cells and leukocytes and released mediators influencing vein wall remodeling, which is analogous to the changes occurring in varicose veins [4].

It is believed that neutrophils play a significant role in the pathogenesis of varicose veins. Strong adhesion of the neutrophils to the vein wall is associated with the interaction of integrins with their specific ligands, which include the intercellular adhesion molecules (ICAMs) on endothelial cells. Neutrophil activation leads to the release of chemokines and cytokines, as well as other processes such as phagocytosis, the exocytosis of intracellular granules, and the release of extracellular neutrophil traps (NETs) and reactive oxygen species (ROS) [5]. Although neutrophil activation is associated with pathogen killing, it can also lead to tissue damage in autoimmune and inflammatory diseases.

In response to the overproduction of ROS, oxidative stress (OS) occurs, which is a consequence of an imbalance between the production of reactive oxygen species and their removal. Oxidative stress can lead to inflammation and the development of many diseases, including cardiovascular diseases. Additionally, OS increases inflammation. It has been shown that leukocytes trapped in vessel walls are capable of releasing ROS. In patients diagnosed with CVD, depending on the clinical condition according to the CEAP (Clinical-Etiology-Anatomy-Pathophysiology) classification, a higher concentration of malondialdehyde (MDA) is observed. An increase in the level of MDA, as measured by the lipid peroxidation index, correlated with a simultaneous decrease in superoxide dismutase (SOD) activity and a decrease in antioxidant potential compared to normal veins [6]. Higher levels of thiobarbituric acid-reactive substances (TBARSs) have been demonstrated in varicose veins with superficial thrombophlebitis compared to normal saphenous veins. Moreover, a significantly higher activity of myeloperoxidase (MPO) and xanthine oxidase (XO) was found. The increase in these parameters indicates the occurrence of oxidative stress in varicose veins [7]. Increased markers of oxidative stress, e.g., higher levels of thiobarbituric acid-reactive substances (TBARSs) and carbonyl compounds were also found in the plasma of patients with varicose veins [8]. In addition to an increase in plasma MDA levels in patients with ineffective venous valves (venous reflux), an increase in the expression of genes and proteins such as iNOS (inducible phagocytes) and NOX2 (NADPH oxidase 2, also known as cytochrome b(558) subunit beta) was observed. Both oxidases are capable of producing large amounts of superoxide, a precursor to other ROS [9].

This review discusses the production of ROS, the pathogenesis of varicose veins as a pathology based on hypoxia, inflammation, and oxidative stress, as well as the participation of red blood cell this disease.

## 2. Reactive Oxygen Species

**Figure 1:**
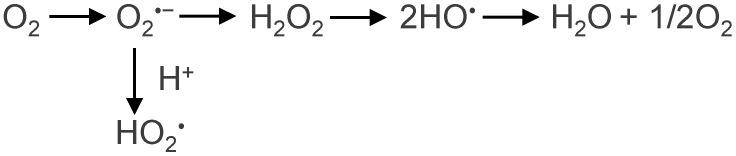
Reduction of molecular oxygen.

Reactive oxygen species are formed as a result of the reduction of oxygen. One-electron reduction of O_2_ leads to superoxide anion (O_2_^•−^), a precursor to other ROS. As a result of a two-electron reduction of oxygen, hydrogen peroxide (H_2_O_2_) is formed, and a three-electron reduction produces a hydroxyl radical. However, a four-electron reduction leads to the formation of a water molecule (Figure 1).

Single-electron reduction leads to a superoxide anion (O_2_^•−^). This process occurs in mitochondria and/or is catalyzed by xanthine oxidase, NADPH oxidase, or NOX oxidase. O_2_^•−^ can undergo spontaneous dismutation or/and be catalyzed by SOD to hydrogen peroxide (H_2_O_2_). The formation of hydrogen peroxide can also occur under the influence of NADPH oxidase 4 (Nox4). H_2_O_2_ is decomposed by catalase (Cat), peroxiredoxin (Prx), and glutathione peroxidase (Gpx) into water and molecular oxygen. In turn, the one-electron reduction of H_2_O_2_ catalyzed by transition metal ions (Fe, Cu, Ti, Co, Cr, Mn) leads to the hydroxyl radical. These reactions take place in the electron transport chain in mitochondria, with the participation of enzymes, iron-sulfur clusters, and others, and their main goal is to produce ATP [10].

There are at least 11 different sites in mammalian mitochondria related to substrate catabolism and the electron transport chain that can produce superoxide and/or hydrogen peroxide. These key substances can be produced in large quantities in the mitochondrial matrix and the cytosolic side of the inner mitochondrial membrane [11]. A total of 0.6% of the superoxide anion radical occurs in the protonated form as the hydroperoxyl radical HO_2_^•^, which shows much higher reactivity compared to O_2_^•−^. During uncatalyzed dismutation, the highest reaction rate constant (k = 4 × 10^9^ M^−1^ s^−1^) is observed during the reaction of hydroperoxide with the superoxide anion radical and the lowest during the dismutation of two superoxide anion radicals (k < 0.3 M^−1^ s^−1^) [12].

$${2HO}_{2}^{•}+O_{2}^{•-}\to H_{2}O_{2}+{2O}_{2}$$

$${2H}^{+}+O_{2}^{•-}+O_{2}^{•-}\to H_{2}O_{2}+O_{2}$$

Of course, superoxide can also be formed in other reactions, such as the oxidation of hypoxanthine to xanthine and xanthine to uric acid with the participation of xanthine oxidase. In turn, hydrogen peroxide is formed as a result of spontaneous (k = 8 × 10^4^ M^−1^ s^−1^) or SOD-catalyzed dismutation (k = 2 × 10^9^ M^−1^ s^−1^), but many other enzymes generate H_2_O_2_, e.g., NOX4. Hydrogen peroxide is rapidly decomposed by catalase (k = 0.6 × 10^7^ M^−1^ s^−1^), peroxiredoxin (Prx-2) (k = 1 × 10^8^ M^−1^ s^−1^), and glutathione peroxidase (k = 4.1 × 10^7^ M^−1^ s^−1^) into water and molecular oxygen. On the other hand, the one-electron reduction of H_2_O_2_ catalyzed by transition metal ions (Fe, Cu, Mn, Co, Ni, Ti, V, Ce, Cr) in the Fenton reaction (Fe) and/or Haber–Weiss reaction, respectively, leads to a hydroxyl radical [13].

$${Fe}^{2+}+H_{2}O_{2}\to {Fe}^{3+}+{HO}^{•}+{HO}^{-}\;O_{2}^{•-}+H_{2}O_{2}\to O_{2}+{HO}^{•}+{HO}^{-}$$

The disintegration of the O–O bond in the H_2_O_2_ molecule requires little energy, which in the presence of a suitable catalyst is approximately 48.75 ± 0.005 kcal/mol [14].

The hydroxyl radical is also formed during the decomposition of hydrogen peroxide initiated by UV radiation quanta.

$$H_{2}O_{2}\to {2HO}^{•}$$

The hydroxyl radical (HO^•^) is one of the strongest oxidizing agents [15]. Diffusion-controlled reaction rates with molecules and macromolecules have rate constants of 10^9^–10^10^ M^−1^ s^−1^ [16]. The HO^•^ radical initiates damage to macromolecules such as proteins, DNA, and lipids [17]. Transition metals also catalyze the decomposition of peroxides and hydroperoxides yielding alkoxyl radicals [18].

$$RO\text{-}OR\to {RO}^{•}+{RO}^{-}\;RO\text{-}OH\to {RO}^{•}+{HO}^{-}$$

Both reactions are important during lipid peroxidation initiating the breakdown of subsequent lipid molecules. Peroxides and hydroperoxides undergo homolytic disintegration of the O–O bond, producing pairs of free radicals [19].

$$RO\text{-}OR\to {RO}^{•}+{RO}^{•}\;RO\text{-}OH\to {RO}^{•}+{HO}^{•}\;RO\text{-}OH\to {ROO}^{•}+H^{+}\;RO\text{-}OH\to {RO}^{•}+{HO}^{-}$$

As a result of the catalytic decomposition of alkyl hydroperoxides, peroxyl radicals (ROO^•^) and alkoxyl radicals (RO^•^) are formed [20]. Both types of radicals have strong oxidizing properties and are formed during the decomposition of peroxides in the presence of transition metals and also during the action of UV radiation. A classic example of the presence of both radicals is the process of the oxidation of polyunsaturated fatty acids (PUFA) during lipid peroxidation. Both radicals actively participate in the initiation stage associated with separating the atom hydrogen in the methylene group adjacent to the double bond in subsequent PUFA molecules.

Another oxidizing agent, singlet oxygen [^1^O_2_, O_2_(1Δg)], has a half-life of 10^−6^ s [21]. Although it is rarely found in biological systems, it can be produced in various reactions in vivo [22]. Singlet oxygen, unlike triplet oxygen, has strong oxidizing properties and easily initiates protein oxidation, nucleic acids, and lipids. It has been shown that the lifetime of ^1^O_2_ in the cell is relatively long and it can diffuse over considerable distances, penetrating cell membranes into extracellular spaces [23]. Singlet oxygen is also generated by the reaction of superoxide anion with glutathione [24]. The reactivity of singlet oxygen is higher than the superoxide anion radical. It can initiate the oxidation reactions of macromolecules such as proteins, nucleic acids, and lipids, either by direct reaction or by induction of ROS. Usually, singlet oxygen is obtained from oxygen in the presence of sensitizers. Excitation of the sensitizer (dye) with a radiation quantum causes its excitation in the singlet state and transition to the triplet state:

sens+hv1→sens*1→sens*3

The excited sensitizer in the triplet state reacts with molecular oxygen (triplet state), yielding singlet oxygen:

sens*3+O2→sens*1+O21

Photosensitizers include anthraquinone derivatives (hipericin), phenothiazine (methylene blue), xanthanea (rose bengal), and merocyanine 540 [25]. The endogenous photosensitizer is protoporphyrin IX. Singlet oxygen generation is used in photodynamic therapy (PDT) in the treatment of cancers such as leukemia, neuroblastoma, basal cell carcinoma, adenocarcinoma, bladder carcinoma, cervical tumor cells, and others. In turn, the potential in vivo sources include the reaction of hypochlorites with hydrogen peroxide [26].

H2O2+ClO−→O21+Cl−+H2O

Hypochlorites are formed during the oxidation of chlorides by H_2_O_2_ in the presence of myeloperoxidase, which occurs in neutrophils [27].

$$H_{2}O_{2}+{Cl}^{-}\to {ClO}^{-}+H_{2}O$$

Moreover, hypobromous acid (HBrO) is similarly formed when bromides are the oxidized substrate. While the myeloperoxidase present in the granules of polymorphonuclear leukocytes (PMNs) catalyzes the oxidation of chlorides and bromides to the corresponding acids, HClO and HBrO, the eosinophil peroxidase (EPO) contained in the granules of eosinophils only catalyzes the oxidation of bromides [28]. Both acids are an important line of defense in inflammation because they kill pathogens. Moreover, hypochlorous acid is highly reactive and has strong oxidizing properties. In reaction with Fe^2+^, it generates a hydroxyl radical. These reactions can only occur locally when iron ions are available. Interestingly, this reaction is faster than with hydrogen peroxide (Fenton’s reaction) and can be a source of hydroxyl radicals [29].

$$HClO+{Fe}^{2+}\to {HO}^{•}+{Fe}^{3+}+{Cl}^{-}\;HClO+{Fe}^{3+}\to {HO}^{-}+{Fe}^{2+}+{Cl}^{•}$$

In turn, the reaction with Fe^3+^ ions leads to the formation of free chlorine atoms, which with water form a hydroxyl radical.

$${Cl}^{•}+H_{2}O\to {HO}^{•}+H^{+}+{Cl}^{-}$$

These reactions can only occur locally when iron ions are available [29]. Hypochlorous acid also reacts with the superoxide anion radical similarly to hydrogen peroxide, generating a hydroxyl radical [30,31].

$$HClO+O_{2}^{•-}\to {HO}^{•}+{Cl}^{-}+O_{2}$$

Another source of singlet oxygen is the recombination of the peroxyl radical according to the Russell mechanism [32].

R1R2CH-O-O•+R1R2CH-O-O•→R1R2CH-O-O-O-O-CHR1R2→O21+R1R2C=O+R1R2CH-OH

Singlet oxygen can also be formed during the decomposition of peroxynitrite (ONOO^−^) [33].

ONOO−+ONOOH→O21+2NO2−

Singlet oxygen can also be formed with the participation of an oxygenase, such as lipoxygenase [34]. It has been shown that singlet oxygen can also be generated in the Haber–Weiss reaction [35].

The vascular endothelium produces nitric oxide (^•^NO), which is a colorless, paramagnetic gas synthesized in the body from L-arginine with the participation of nitric oxide synthases (NOS), oxygen, and cofactors such as NADPH and tetrahydrobiopterin. Nitric oxide modulates the tone of blood vessels, peristalsis, insulin secretion, neural development, and angiogenesis. Since it is a very small and uncharged molecule, it can easily diffuse through biological membranes and quickly travel long distances [36]. Nitric oxide does not exhibit strong oxidizing or reducing properties and is chemically passive toward most biomolecules.

However, in the presence of oxygen, nitrogen oxide is oxidized to paramagnetic nitrogen dioxide (^•^NO_2_), which is a reddish-brown gas with a sharp, unpleasant odor.

NO•+12O2→NO2•

As a radical, ^•^NO reacts rapidly with other radicals, such as O_2_^•−^, HO^•^, tyrosyl (Tyr^•^), thiyl (RS^•^), ^•^NO_2_, and superoxide (ROO^•^) [37]. In addition, ^•^NO_2_ is highly toxic and has strong oxidizing and nitrating properties [38]. In the body, it initiates the process of lipid peroxidation and tyrosine residue nitration in proteins. The reaction of ^•^NO_2_ with ^•^NO produces nitrous acid anhydride (N_2_O_3_), which reacts with thiols to yield nitrosothiols RSNO and water to form nitrous acid [39]. In turn, amines are also nitrosated to form nitrosamines. The agent that nitrosates thiols and amines is the nitrosonium cation NO^+^. In the diffusion-controlled reaction of nitric oxide with superoxide anion, the peroxynitrite ion (ONOO^−^) is formed. The high reaction rate constant (k = 4–16 × 10^9^ M^−1^s^−1^) makes this reaction competitive with the dismutation of the superoxide anion radical catalyzed by SOD and the diffusion of ^•^NO across cell membranes.

NO•+O2•−→ONOO−

Peroxynitrite is a strong oxidizing and nitrating agent that is associated with the decomposition of peroxynitrous acid (ONOOH, pKa = 6.8). ONOOH decomposes to form 70% nitric acid, while 30% of it forms a complex consisting of the hydroxyl radical and nitrogen dioxide (caged radical pair) [40,41]. The main targets of ONOO^−^ action are thiols such as glutathione and cysteine. Since the concentration of glutathione (5–10 mM) in cells is much higher than that of cysteine, it is the main scavenger of peroxynitrite. ONOO^−^ also shows high affinity for selenoproteins, such as GPx, which participates in protecting the cell from peroxynitrite, as well as 4Fe-4S clusters. The reaction of peroxynitrite with the 4Fe-4S cluster leads to the inactivation of aconitase. Similarly, it inactivates alcohol dehydrogenase by acting on the zinc–sulfur center. In addition, it reacts with the heme centers in hemoglobin, cytochrome c2, and peroxidases with reaction rate constants of 10^4^–10^6^ M^−1^ s^−1^ [40]. Peroxynitrite creates nitro derivatives with tyrosine and tryptophan residues of proteins and guanine residues in DNA as well as with aliphatic fatty acids and sugars.

ONOOH→NO3−+HO•⋯NO2•

In the presence of carbon dioxide, which is present in the body, peroxynitrite forms a strongly oxidizing complex composed of nitrogen dioxide and a carbonate anion radical (caged radical pair) [41].

ONOO−CO2→NO2•⋯OCO2−•→NO2•+⋯OCO2−•

The presence of carbon dioxide affects the reactivity of peroxynitrite. While the carbonate radical anion has much lower reactivity than the hydroxyl radical, its presence influences the greater nitrating properties of peroxynitrite [41].

In addition, quinones and hydroquinones occur in cells and tissues as products of their two-electron reduction. However, the one-electron reduction of quinones and the one-electron oxidation of hydroquinones lead to semiquinone radicals.

$$Q\to Q^{•-}\to {QH}_{2}$$

Benzoquinone and naphthoquinone derivatives are reduced by flavoproteins to semiquinones, and their reaction with oxygen leads to the production of superoxide anion radicals [42].

$$Q^{•-}+O_{2}\to Q+O_{2}^{•-}$$

Semiquinones are reactive and can form adducts with biomolecules such as proteins, lipids, and DNA [43,44,45].

ROS are generated in mitochondria (electron transport chain, I, II, III), in plasma membranes (NADPH oxidase, lipoxygenases, quinone oxidase), in the endoplasmic reticulum (flavoproteins, cytochrome P450), and in the cytoplasm (xanthine oxidase, NOS isoforms) [46]. In the conditions of body homeostasis, ROS act as regulators, mediators, and signaling molecules in numerous reaction cycles that occur in cells and tissues. Over 40 enzymes, and other molecules such as nitric oxide, hydrogen sulfide, and oxidized lipids, participate in the redox signaling process [47]. Moreover, they influence the course of the proper immune response, aging processes, and cell death [48]. Moreover, ROS can also be generated in the body by external factors, such as some xenobiotics, metals, a, b, g radiation and X-rays, UV radiation, smoking, and others. Also, many anticancer drug derivatives of anthracyclines (doxorubicin) and anthraquinones (mitoxantrone), but also cyclosporin, bleomycin, and cisplatin, generate reactive oxygen species. Other drugs that produce ROS include hydralazine (relaxes blood vessels), diclofenac (anti-inflammatory), paracetamol (analgesia), and others [49].

## 3. Antioxidant Systems

Cells and tissues have an arsenal of diverse defense mechanisms that inactivate reactive oxygen species. Their purpose is to protect vital biological molecules from damage. The defense systems include enzymes such as superoxide dismutase (SOD), catalase (Cat), glutathione peroxidases (GPx), peroxiredoxins, thioredoxins, glutaredoxins, and others.

### 3.1. Antioxidant Enzymes

Superoxide dismutases catalyze the dismutation of the superoxide anion radical. In the cytosol and plasma, zinc-copper superoxide dismutase (CuZnSOD, SOD1, and SOD3) is present, while in the mitochondria, manganese superoxide dismutase (MnSOD, SOD2) is present. Superoxide dismutase CuZnSOD has a Cu^2+^ ion in its active center, which is reducible by superoxide to a Cu^+^ ion and the product is molecular oxygen. Another superoxide anion radical molecule oxidizes the Cu^+^ ion to a Cu^2+^ ion and the product is hydrogen peroxide. Manganese dismutase works in a similar way, where the manganese ion (Mn^3+^/Mn^2+^) is reduced/oxidized by the superoxide anion radical. The rate constant is high and equal to k = 2 × 10^9^ M^−1^ s^−1^. CuZnSOD also exhibits peroxidase properties, breaking down hydrogen peroxide. However, the reduced Cu^+^ ion reacts with H_2_O_2_, forming a hydroxyl radical that inactivates the enzyme. Additionally, zinc copper dismutase forms a complex with peroxynitrite, which nitrates tyrosine residues.

The toxic hydrogen peroxide produced by the dismutation of superoxide anions is broken down into water and oxygen by catalase, peroxidases, and peroxiredoxins. Catalases in the active center have a heme system with an iron ion Fe^3+^ during the reaction with hydrogen peroxide; the iron is oxidized to the radical ferryl form (Fe^4+^=O^2−^)^•+^, the π-cation of the porphyrin, also called Compound I [50]. This arrangement occurs in all oxidized forms of peroxidases and catalases. A second molecule of hydrogen peroxide reduces the ferryl form to the enzyme’s parent form with Fe^3+^. Another enzyme that breaks down hydrogen peroxide is GPx. There are currently eight different glutathione peroxidases known to occur in different organs [51]. Unlike catalase, which is found in peroxisomes, GPx1 is located in the cytosol. The GPx described by Mills contains selenocysteine (-SeH) in its active center, which is oxidized by hydrogen peroxide or organic peroxides to selenic acid (I) (-Se-OH) [52]. The latter is formed from GSH, a selenium–sulfur transition bridge (-Se-S-G), which is reduced by another GSH molecule to the active form of the enzyme (-SeH) and GSSG. Importantly, the rate constant of the GPx reaction with H_2_O_2_ is one to two orders of magnitude higher than that of catalase. Because glutathione is a substrate for GPx, it is oxidized to GSSG. The reduced form (GSH) is restored by glutathione reductase, for which the substrate is NADPH. Glutathione peroxidases are irreversibly inactivated by cyanide [53].

Peroxiredoxins (Prxs) are a family of cysteine-dependent enzymes that are widely distributed in the body and whose main function is to regulate the concentration of superoxide in cells. Since the removal of toxic H_2_O_2_ is crucial, the enzymes are present in large quantities. At the active site of the enzyme, cysteine thiolate (Cys-S-) is oxidized to CP-sulfenic acid (Cys-SOH) and then reduced to dithiol. The final step is the reduction of the dithiol by thioredoxin to the active form of the enzyme [54]. Prxs were initially thought to be three orders of magnitude slower in action than catalases and peroxidases. However, recent studies have shown that their reaction rate constant is k = 10^7^–10^8^ M^−1^ s^−1^. Catalase and peroxiredoxin-2 have been shown to react with hydrogen peroxide at comparable rates. Prx-2 reacts with a rate constant of 1.3 × 10^7^ M^−1^ s^−1^ [55].

In terms of glutaredoxins (Grx), five isoforms are small proteins with two residues of cysteine in the active site. Grx catalyze glutathione-dependent glutathionylation, i.e., the coupling of GSH to the substrate, as well as the reverse reaction (deglutathionylation). In addition, Grx use the reducing power of GSH to catalyze the reduction of dithiols in the presence of NADPH and glutathione reductase (glutaredoxin system) [56]. Because Grx has thiol-redox properties, it also impacts the activity of dehydroascorbate reductase and transhydrogenase. Additionally, it participates in denitrosylation and the reduction of cystine to cysteine. Grx also has thioltransferase activity and has a role in iron homeostasis, which is an important function in living organisms. In addition, cells have low molecular weight antioxidants, including glutathione, cysteine, ascorbic acid, tocopherols, carotenes, lipoic and dihydrolipoic acids, uric acid, and others.

In a normally functioning organism, the level of reactive oxygen species is low, which is related to maintaining a balance between the release of ROS and their removal. ROS participate in cell differentiation and migration but also in apoptosis and cell necrosis. They are also important in the physiology of the organism, redox homeostasis, and the regulation of transcription factors. However, in disease states or as a result of the effects of various external factors, the level of ROS can be significantly higher. Additionally, there may also be a weakening of antioxidant systems, the inefficiency of which can lead to oxidative stress; the consequence of which is damage to important life molecules such as proteins, lipids, and nucleic acids [57]. On the other hand, cells can activate systems that repair damage. However, even in this case, their activity may be limited. If, for example, damaged DNA molecules are not repaired, this can lead to mutations, rearrangements, and transcription errors that affect important components of mitochondria, which ultimately leads to increased oxidative stress [58].The most important enzymatic and low molecular weight antioxidants are presented in Table 1.

### 3.2. Low Molecular Weight Antioxidants

Endogenous low molecular weight antioxidants (LMWAs) can be divided into those soluble in the water environment, cytosol, and lipid environment (biological membranes). The first group includes GSH and ascorbic acid and the second tocopherols, carotenes, and quinones. An important feature of LMWA is the reaction with free radicals, which leads to the termination of chain reactions. In addition, many antioxidant substances, such as phenols (e.g., gallic acid, caffeic acid), diphenols (resveratrol), a wide spectrum of flavonoids (flavan, flavanones, isoflavans, neoflavans, flavones, isoflavones, flavalyl ion, neoflavones), and others, enter the body with food.

### 3.3. Thiols

The thiol groups present in amino acids, peptides, proteins, and enzymes are sensitive to oxidizing agents. Their presence is also key in the function of many proteins/enzymes, influencing the functional and structural properties of these biomolecules. The presence of thiols helps maintain redox homeostasis in cells, protects against oxidizing agents, heavy metals, and xenobiotics, and also participates in redox signaling [59]. Thiols also directly participate in scavenging reactive oxygen species, including free radicals.

$$R^{\prime }SR+R^{•}\to {R^{\prime }S}^{•}+RH$$

Thiol radicals react with the thiolate anion to form the corresponding anion radicals:

$${R^{\prime }S}^{•}+{RS}^{-}\to {R^{\prime }S-SR}^{•-}$$

The latter reacts with molecular oxygen to form superoxide anion radicals and dithiols (disulfides):

$${R^{\prime }S-SR}^{•-}+O_{2}\to O_{2}^{•-}+R^{\prime }S-SR$$

On the other hand, thiols have been shown to scavenge the superoxide anion radical and hydrogen peroxide [60,61,62]. For example, the reaction of GSH with the superoxide anion radical requires the presence of molecular oxygen [61,63].

$$RSH+O_{2}^{•-}+H^{+}\to {RS}^{•}+H_{2}O\;{RS}^{-}+H_{2}O_{2}\to RSOH+H_{2}O$$

Thiols reduce the resulting sulfinic acid to dithiols [64].

$$RSOH+R^{\prime }SH\to RS-SR^{\prime }+H_{2}O$$

A very important thiol (tripeptide) occurring in cells is glutathione (GSH). Its concentration in the cytosol can be 0.5–11 mM. A similar concentration of GSH is present in mitochondria and its concentration can be higher in the cell nucleus (3–15 mM) [65]. High concentrations of reduced glutathione in the cytosol allow for maintaining strongly reducing conditions, which protects cell components from oxidation. The formation of dithiol bonds in the cytosol is precisely regulated and related to the signaling function. In contrast, the unpredictable oxidation of -SH groups to -S-S- bridges is reversed by thioredoxin. In turn, the oxidized form of thioredoxin is reduced by thioredoxin reductase. This system works independently of the glutathione-related systems. Additionally, protein dithiols can also be reduced by glutaredoxin (Grx). The resulting -S-S- bridges in Grx are reduced by GSH. Glutathione is also a substrate for many enzymes, including glutathione peroxidase. GSH reduces the oxidized form of glutaredoxin to the active reduced form [66].

Other antioxidant thiols, lipoic acid (LA), and dihydrolipoic acid (DHLA) are effective scavengers of hypochlorous acid. Moreover, LA was a strong hydroxyl radical scavenger, inhibiting the lipid peroxidation initiated by Fe^3+^ ions in the presence of ascorbate. In contrast to LA, DHLA accelerated the lipid peroxidation initiated by Fe^3+^ ions, probably by reducing them to Fe^2+^ ions. On the other hand, DHLA did not accelerate lipid peroxidation via the myoglobin/hydrogen peroxide system. Interestingly, both acids did not react with either the superoxide anion radical or hydrogen peroxide [67].

Usually, the redox properties of proteins possessing thiol groups are associated with the formation of disulfide bridges (-S-S-); however, in other proteins, -SH groups are oxidized to sulfenic acids (RCys-SOH). While the formation of sulfenic acids is a reversible reaction, the formation of more oxidized (higher oxidation state of the sulfur atom) derivatives, such as sulfinic acids (RCys-SO_2_H) and sulfonic acids (RCys-SO_3_H), is an irreversible reaction. Cys-SO_2_H and sulfonic acids (RCys-SO_3_H) are formed during oxidative damage to proteins. The formation of more oxidized forms of sulfur, i.e., sulfinic and sulfonic acids, was observed during myocardial ischemia and reperfusion [68].

### 3.4. Ascorbic Acid

Ascorbic acid (vitamin C, Asc) is the second most important low molecular weight antioxidant found in the cytosol. It occurs in an ionized form, i.e., ascorbate anion (Asc^−^), pKa = 4.17. Its one-electron oxidation leads to the ascorbyl radical (Asc^•−^). An example is the reaction with free radicals, in which ascorbate is a scavenger. Reaction with another radical leads to dehydroascorbate [69].

$${Asc}^{-}+R^{•}\to {Asc}^{•-}+RH\;{Asc}^{•-}+R^{•}\to DHAsc+RH$$

Ascorbate reacts with the superoxide radical with a rate constant of k = 1 × 10^5^ M^−1^ s^−1^ but as an ascorbyl radical with a rate constant of 2.6 × 10^8^ M^−1^ s^−1^. The highest rate constants are for hydroxyl and alkoxy radicals, k = 1.1 × 10^10^ M^−1^ s^−1^ and k = 1 × 10^9^ M^−1^ s^−1^, respectively. Lower rate constants are found for superoxide and α-tocopheryloxyl radicals, k = 10^6^ M^−1^ s^−1^ and k = 10^7^ M^−1^ s^−1^, respectively [69].

Although ascorbate is an effective antioxidant, it exhibits pro-oxidant activity under certain conditions. This involves the single-electron reduction of transition metals, such as Fe^3+^, Cu^2+^, and others, occurring in higher oxidation states, which become catalysts in the Fenton- and/or Haber–Weiss-type reactions. Ascorbate is involved in the action of numerous enzymes, such as procollagen-proline, procollagen-lysine dioxygenase, dopamine b-monooxygenase, ascorbate oxidase, ascorbate peroxidase, and cyt *b*5 reductase [69].

### 3.5. Tocopherols

The next antioxidants present in the lipid part of the membrane are the tocopherols α, β, γ, and δ, differing in the number of methyl substituents in the chroman ring. The predominant tocopherol is α-tocopherol (vitamin E, α-TOH), which, similarly to other tocopherols, protects lipids from the peroxidation initiated by reactive oxygen species. As a result of the capture of free radicals initiating the lipid peroxidation process, such as the HO^•^ radical or secondary radicals RO^•^ and ROO^•^ reinitiating the peroxidation process, the α-tocopheryloxyl radical (α-TO^•^) is formed [70]. This radical is captured by ascorbate, transforming into active vitamin E. The tocopheryloxyl radical can also be regenerated by flavonoids [71].

### 3.6. Carotenoids

Carotenoids are another group of antioxidants soluble in the lipid environment. Among the numerous compounds found in vegetables and fruits, we can include α, β-carotene, lutein, lycopene, zeaxanthin, and others. β-Carotene is mainly found in biological membranes. Carotenoids effectively scavenge not only the radicals involved in lipid peroxidation, such as HO^•^, RO^•^, ROO^•^, RO^•^, but also O_2_^•−^ and nitrogen dioxide. The reaction rate constants are high, e.g., for peroxide radicals it is k = 10^8^ M^−1^ s^−1^ [72,73].

$$\beta \text{-}Car+R^{•}\to {\beta \text{-}Car}^{•+}+RH$$

α-Tocopherol regenerates carotene cation radicals to the original β-carotene [72].

$${\beta \text{-}Car}^{•+}+\alpha \text{-}TOH\to \beta \text{-}Car+{\alpha \text{-}TO}^{•}+H^{+}$$

**Figure 2:**
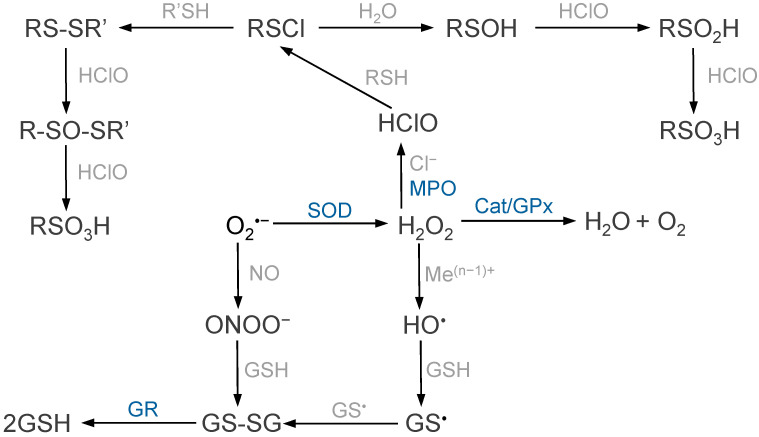
Decomposition of reactive oxygen species by enzymes and low molecular weight antioxidants. While ROS resulting from the reduction of molecular oxygen are removed by enzymatic systems (SOD, Cat, GPx), the hydroxyl radical, hypochlorous acid, and peroxynitrite can react directly with GSH to form oxidized GSSG. GSSG is reduced to GSH by glutathione reductase.

The main protective strategy of carotenoids is associated with, e.g., protection against lipid peroxidation, preventing the formation of radicals initiating the process. In the case of the HO^•^, in addition to endogenous low molecular weight oxidants, transition metal ion chelators are used, and in the case of O_2_^•−^, inhibitors of enzymes generating this radical are used, e.g., allopurinol in the case of xanthine oxidase. To inhibit chain propagation, it is important to interrupt chain reactions in which LMWAs, mainly tocopherols and carotenes, participate and to accelerate the termination stage with the participation of all low molecular weight antioxidants (Figure 2).

## 4. Varicose Vein

Chronic venous disease (CVD) is a disease of the veins of the lower limbs. In varicose veins (VVs), the normal structure of the vein is altered, with changes in the thickness and composition of the venous wall [74]. The veins become deformed and develop into VVs, which are dilated, bulging, twisted veins just beneath the skin’s surface. Varicose veins occur quite often. The risk of VVs and CVI is higher in women (2.6%) than in men (1.9%) and increases with age, which makes for a high possibility of occurrence and an increasing incidence [1]. Using the CEAP classification, most patients (70%) can be assigned to classes C0 and C1, while about 25% of patients are diagnosed with VV (C2). In turn, CVI (C3–C6) accounts for about 5% and is a small percentage of patients with CVD [75].

The main factors occurring in the pathology of varicose veins are hypoxia and chronic inflammation [2]. In addition, blood flow disorders cause the excessive release of various inflammatory factors that activate coagulation processes. The formation of varicose veins leads to dysfunction of the venous endothelium and causes a decrease in the biosynthesis of nitric oxide, an important signaling molecule and inhibitor of platelet adhesion and aggregation, as well as the adhesion of leukocytes to endothelial cells [76]. Additionally, stimulated leukocytes release cytokines, ROS, and proteolytic enzymes and platelet-activating factor. In the blood of varicose veins, an increase in the level of the cytokines IL-6 and IL-8 was demonstrated, which indicates an inflammatory state [77]. It is postulated that the cytokines IL-6, IL-8, and IL-1b are involved in whole-blood hypercoagulation, RBC damage, and increased platelet activation, which leads to the formation of vascular clots. The changes in the shape of RBCs initiated by IL-8 are similar to those observed in eryptosis [78]. In addition, a relationship between varicose veins and mean corpuscular hemoglobin concentration (MCHC) has been demonstrated, which is evidence of the effect of hemoglobin on varicose veins [79].

Smooth muscle cells (SMCs) of the venous wall are an important element in vascular homeostasis. Changes in SMCs can affect the increase in endothelial permeability and the release of various substances. SMCs from VVs are more dedifferentiated and are characterized by greater migration, proliferation, and release of MMP-2 [80]. Venous insufficiency is a cause of cellular hypoxia that disrupts homeostasis [81].

Untreated varicose veins can develop into chronic venous insufficiency (CVI), in which venous leg ulcers (VLU) appear. The development of varicose veins is related to family and genetic factors, but environmental, behavioral, and dietary factors are also important. The disease is promoted by a sedentary lifestyle, overweight or obesity, older age, being female, pregnancy and some contraceptive drugs, and smoking. Changes in mRNA expression, protein levels, and the proteolytic activity of matrix metalloproteinases (MMPs) have been demonstrated in varicose veins. An imbalance of matrix metalloproteinases (MMPs) and their inhibitors (TIMPs) contributes to the development of the disease. MMP expression may be caused by a higher hydrostatic pressure in veins; additional factors are the hypoxia caused by blood stasis and venous reflux, as well as tissue metabolites and inflammation. Increased MMP activity increases the proteolysis of various proteins in the extracellular matrix, this mainly concerns collagen and elastin, and weakens the vein wall. The consequence of MMP expression may be venous dilation through the increased release of vasodilating factors from endothelial cells, which causes hyperpolarization and relaxation of smooth muscles [82].

Veins have bicuspid venous valves that determine the flow of blood in the right direction and prevent venous reflux [83]. In addition, muscle pumps work synchronously with the venous valves, allowing blood to flow in only one direction. Damage to the valves and disorders in the functioning of muscle pumps lead to venous reflux and venous stasis and, consequently, to hypoxia [84,85].

### 4.1. Role of Endothelium

The endothelium is a single layer of cells that lines the inside of the vessels. This layer regulates most of the functions of blood vessels. In response to stimulating factors, there is a change in vascular tone and inflammation, which is accompanied by the recruitment of the immune system and angiogenesis. If any of these functions are disturbed, then the endothelium becomes dysfunctional [86]. Research has shown that reactive oxygen species are of key importance in each of the endothelium’s roles in sensing/signaling and endothelial dysfunction. Because endothelial cells are a layer separating the blood from the vessel wall, they are important not only in vascular homeostasis but also in the adaptation of vessels to environmental changes. The endothelium is key in the regulation of blood flow, vascular permeability, inflammatory cell recruitment, thrombosis, and angiogenesis. Disturbance of its structure and function affects vascular homeostasis. The vascular endothelium can produce reactive oxygen species. NADPH oxidases (NOX) are the major source of superoxide and hydrogen peroxide production in the endothelium. The NOX family includes NOX1, NOX2, NOX3, NOX4, NOX5, and DUOX1 and 2. NOX1, 2, 4, and 5 are found in cells of the vasculature. NOX1, NOX2, and NOX4 are probably located in the endothelium. NOX2, which is located in the EC membrane, is an enzyme that produces large amounts of superoxide in response to a change in shear stress. However, O_2_^•−^ is dismutated spontaneously or by SOD, so H_2_O_2_ becomes the main factor in signal transduction in the signaling cascade mediated by reactive oxygen species, like O_2_^•−^, that are produced outside the cell. Similarly, an increase in NOX4 expression occurs in response to a change in shear stress. Therefore, a change in shear stress causes an increase in ROS production by NOX4 and/or NOX1 in endothelial cells (ECs) [87,88]. Moreover, ROS production by NADPH oxidase can be stimulated not only by shear stress but also by environmental factors (e.g., hypoxia) and cytokines and hormones, such as angiotensin II, aldosterone, endothelin-1 (ET-1), growth factor (PDGF), transforming growth factor β (TGFβ), and tumor necrosis factor α (TNFα) [88].

^•^NO is produced by endothelial NOS synthase (eNOS) as a result of various stimuli, such as, for example, increased shear stress or the activation of G protein-coupled receptors by thrombin or acetylcholine [87]. The increase in ^•^NO production is also associated with a change in shear stress but is dependent on the presence of calmodulin, which indicates the participation of calcium ions in this process. Nitric oxide has a strong affinity for heme proteins. ^•^NO diffuses readily from endothelial cells and interacts with guanylate cyclase to form cyclic GMP (cGMP) from guanosine triphosphate (GTP). cGMP activates protein kinase G, which is involved in protein phosphorylation and causes changes in the dynamics of actin and myosin filaments, leading to smooth muscle relaxation.

The endothelium is important in regulating vascular tone by secreting various relaxing factors, such as nitric oxide (^•^NO), hyperpolarizing factors, and prostaglandins, which dilate blood vessels. In a normally functioning endothelium, ^•^NO is the dominant factor that helps maintain the vascular wall in a state of rest, preventing thrombosis, cell proliferation, and inflammation [89]. In addition, nitric oxide inhibits platelet adhesion and aggregation and also initiates the disaggregation of formed platelet aggregates [90]. In normal endothelium, the ^•^NO released by eNOS inhibits SMC proliferation and has an anti-inflammatory effect [91]. In VVs, an increase in ^•^NO release is observed due to iNOS expression. Additionally, there is an increase in the release of PG12 and EDHF [92].

CVD is a multifactorial disease, but its characteristic features are undoubtedly venous hypertension, valvular insufficiency, and inflammation. Additionally, changes in shear stress cause activation of endothelial cells and consequently the recruitment of leukocytes and release of proinflammatory factors. A dysfunctional endothelium is an important element of the inflammatory cascade, the consequences of which are pathological changes in the vein and the development of chronic venous disease. Importantly, endothelial dysfunction in CVD may be a major factor in the development of deep vein thrombosis [93].

In cultured human varicose vein endothelial cells (HVVECs), we found lower lipid fluidity in the membranes compared to human umbilical vein endothelial cells (HUVECs). We observed changes not only in the subsurface region of the membrane but also in the deeper regions of the lipid monolayer of the plasma membrane. These results indicate that HVVEC plasma membranes are stiffer than those of HUVECs [94]. The fluidity of plasma membranes performs plays an important role not only in the adhesion properties of cells [95,96] but also in cell–cell communication [97,98]. The increased stiffness of HVVEC cell membranes may influence their stronger adhesive properties concerning the other cells involved in the formation of venous thrombus, which is associated with a greater possibility of venous thrombosis.

### 4.2. Inflammation

Endothelial dysfunction is a complex pathophysiological process and may occur under the influence of various inflammatory mediators, such as oxidized plasma lipoproteins, cytokines, and others. Activation of nuclear factor-κB triggers the expression of adhesion molecules, selectins, and chemokines, which contributes to increased adhesion and rolling and, as a result, leukocyte migration to the subendothelial space. An additional factor is shear stress, which causes proinflammatory excitation of the endothelium and affects increased monocyte adhesion and endothelial cell apoptosis. These phenomena are accompanied by the formation of neutrophil extracellular traps and the NLRP3 inflammasome, inflammatory factors that deepen endothelial dysfunction. Inflammation, hypoxia, hypertension, and blood stasis lead to neutrophil aggregation and their adhesion to the endothelium, which is referred to as the leukocyte trap [99,100]. The adhesion process of neutrophils can be initiated by the cytokines IL-8, IFN-γ, and TNF-α, as well as PAF (platelet activation factor), active complement complex, and arachidonic acid metabolites [101]. Initially, neutrophil adhesion occurs via P-, E-, and L-selectins and then via the CD11/CD18 complex with the participation of the endothelial intercellular adhesion molecules ICAM-1 and ICAM-2, which leads to strong adhesion [102,103]. Activated neutrophils can also release molecules such as the adhesion-initiating leukotriene LTB4 endothelial leukocytes, NETs, and various types of cytokines (TNF, IL-1β, IL-6, and IL-17) and chemokines (IP-10, MIP1a, MIP1-b, and MCP1) [104,105,106,107]. In addition, activated neutrophils express NADPH oxidase (NOX2), iNOS, and myeloperoxidase, which results in the release of many reactive oxygen species such as O_2_^•−^, H_2_O_2_, HClO, HO^•^, and ^1^O_2_, organic radicals, which are formed during lipid peroxidation, and also NO, which is the precursor of NO_2_ and ONOO^−^ [30,108,109,110]. A compromised cell endothelium secretes inflammatory mediators, which leads to a prothrombotic state by stimulating the coagulation pathway, characterized by, among other things, decreased thrombomodulin expression and increased platelet adhesion and aggregation caused by the release of von Willebrand factor [111].

During inflammation, the endothelium stimulates the expression of cell adhesion molecules (CAMs) on the cell surface, which facilitate the passage of leukocytes from the bloodstream into the inflamed tissues [89]. In addition, vascular cell adhesion molecule-1 (VCAM-1) and intercellular adhesion molecule-1 (ICAM-1) are expressed. These molecules participate in leukocyte adhesion and rolling [112]. The released junctional adhesion molecules (JAM) (JAM-A, JAM-B, and JAM-C) are mediators of leukocyte-EC and platelet-EC interactions [113]. E- and P-selectins have a carbohydrate-recognizing domain on their surface and promote the binding of specific leukocyte surface glycans [114]. Another group released by the endothelium in inflammation are chemokines (CXC, CC, C, and CX3C), which are low molecular weight peptides and act as chemotactic factors in inflammation. Additionally, disrupted flow and/or oxidized plasma lipoproteins initiate the expression of monocyte chemoattractant protein-1 (MCP-1) on the vessel wall. This protein is involved in the recruitment of monocytes through the interaction between CCL2 on the surface of MCP-1 and CCR2 on the surface of monocytes [115]. ROS release by endothelial cells is carefully controlled to ensure only redox signaling [116]. Higher ROS concentrations can lead to inflammation, initiation of macromolecule oxidation, and endothelial dysfunction [117]. Excessive ROS generation by endothelial cells is associated with increased risk factors for CVD, which occurs in diseases such as atherosclerosis, diabetes, metabolic disease, hypoxia, and homocysteinemia [118]. The exclusion of eNOS and increased production of toxic superoxides not only disturb the heart function but also the venous and arterial systems [119].

As a result of endothelial dysfunction, there is a decrease in the synthesis of anti-inflammatory factors and the expression of pro-inflammatory and prothrombotic molecules [120,121]. Venous reflux causes an increase in hydrostatic pressure and, as a result, a decrease in shear stress, which is a regulator of the endothelial activation state [122]. Disturbances in the regulation of the endothelial state promote the deepening of pathological changes in the vein wall and venous valve [123,124]. A decrease in shear rate activates endothelial cells (ECs) and leukocytes through an increase in the expression of adhesion molecules and the infiltration of inflammatory cells into the venous wall and valves, which promotes local inflammation [99]. Changes in normal EC signaling promote the production of inflammatory mediators such as chemokines, cytokines, growth factors, proteases, and others, which intensifies and perpetuates inflammation.

### 4.3. Risk Factors of DVT in Varicose Veins

Increased venous pressure, damage to the venous wall, and genetic factors can cause both CVD and deep vein thrombosis (DVT). Although varicose veins are located beneath the surface of the skin, in more severe cases there is a risk of DVT. CVD, which includes DVT and pulmonary embolism (PE), is common and becoming a serious clinical problem [125]. In DVT, a clot forms in a vein in the lower extremities. The clot itself is not a major problem, but if it breaks off and travels through the circulation to the pulmonary arteries, it can cause a blockage or pulmonary embolism (PE). PE is the most serious complication and may be fatal due to hypoxia and circulatory collapse [126,127].

As already mentioned, although varicose veins are located beneath the skin’s surface, in more severe cases there is a risk of DVT [128]. A recent study showed a four-fold increased risk of DVT in patients with varicose veins [129]. Varicose veins have also been shown to be an additional factor in DVT in patients with high risk factors for venous thromboembolism (VTE) due to cancer or orthopedic surgery [128,130]. Additionally, a complication of clinical varicose vein surgery is DVT. Although the risk of its occurrence is estimated at approximately 1%, the actual risk of DVT is much higher [131].

### 4.4. Hypoxia

Too slow blood flow or its stasis in varices leads to hypoxia, the consequence of which is increased expression of HIF-1α and HIF-2α proteins and HIF target genes. Thus, the HIF pathway may lead to pathophysiological changes in the VV wall. A reduced oxygen concentration may contribute to VV pathogenesis. The increase in HIF-1α and HIF-2α in hypoxia confirms the role of HIFs in the pathogenesis of VVs [3]. An important factor in the development of VVs is reactive oxygen species, although their level during hypoxia is a subject of discussion. In several works, it was shown that the production of ROS, mainly derived from the mitochondrial electron transport chain (ETC), increases not only in normal cells but also in transformed cells, while in other works the authors believe that the level decreases [132,133,134,135,136]. In hypoxia, an increase in the production of superoxide from complex III is observed, which is caused by the HIF-1α signaling system in cells [132]. Under conditions of oxidative stress, the oxidative pentose phosphate cycle (OPPC) is necessary to maintain the reduction potential of cells. In oxidative conditions, including the presence of ROS, OPPC activity increases. In hypoxia, a strong decrease in OPPC activity in cells was observed [134]. Studies using cell culture and ex vivo explants have shown that hypoxia initiates inflammation and activates leukocytes and endothelium, which release factors that influence vein wall remodeling in a similar way to that observed in varicose veins [137]. It appears that expression of HIFs that are involved in the regulation of genes involved in oxygen homeostasis may lead to an increased release of ROS. It has been shown that hypoxia induces iNOS activity, increases prostacyclin PGI2 and cyclooxygenase-2 (COX-2), and increases the production of the antithrombotic eicosanoid in endothelial cells [138]. Incubation of polymorphonuclear cells (PMNs) with endothelial cells in hypoxia led to the release of superoxide anion and the production of leukotriene B4. These results indicate that hypoxia initiates ROS-dependent endothelial cell damage, which is responsible for the activation of neutrophils. This process may also occur in ischemic tissues, exacerbating tissue damage [139].

### 4.5. Red Blood Cells in CVD and the Formation of Vascular Clots

The vascular clot is formed due to interactions between innate immune cells, platelets, red blood cells, and venous endothelial cells [140]. Although initially it was believed that RBCs remain passive in this process, recent studies have shown that they can promote clot formation (red clot) and affect its stability. It turned out that quantitative and qualitative changes in RBCs were associated with an increased predisposition to arterial thrombosis and venous thrombosis. Erythrocytosis and high hematocrit cause the blood to become thicker and more viscous, which causes slower flow through vessels and organs [141]. While the activation of the coagulation cascade by immune cells, platelets, red blood cells, endothelial cells, and fibrin deposition, results in clot formation, the immune cells involved in initiating blood clot formation also express and release fibrinolytic factors to “dissolve” the clot. An increased risk of thrombosis also occurs in patients with thalassemia and spherocytosis, especially after splenectomy [142]. Thrombosis is also common in patients with paroxysmal nocturnal hemoglobinuria, in which the hemolysis of RBCs dependent on complement occurs. An additional risk of thrombosis is red blood cell transfusion, which is not related to longer storage durations for RBCs. At low (venous) shear rates, slow blood flow and the discoidal shape of RBCs favor RBC aggregation, which is arranged in a rouleaux-like pattern, increasing blood viscosity [143]. RBCs can freely bind to the endothelium or subendothelial matrix. They can also bind to other proteins or cells, such as neutrophils and platelets, consequently leading to venous obstruction [144]. In addition, the hemoglobin released from erythrocytes during intravascular hemolysis can bind nitric oxide and enhance adhesive interactions with the vessel wall. Nitric oxide performs an important role in vascular homeostasis, influencing smooth muscle relaxation and vascular tone, and is also responsible for the expression of endothelial adhesion molecules and platelet activation and aggregation [145]. Thrombosis may also be related not only to impaired blood cells but also to the influence of other blood cells and vessels themselves [141]. Higher hematocrit and higher shear rates in straight vessels result in greater platelet adhesion and accumulation on the fragments’ subendothelium and surfaces with exposed von Willebrand factor or collagen [146,147]. Additionally, hemoglobin and ADP released during RBC lysis lead to increased platelet activation and aggregation [145,148]. The released platelet factors stimulated by RBCs increase platelet exposure to P-selectin and αIIbβ3 integrin activation, indicating that RBCs contribute to the procoagulant properties of platelets [149].

### 4.6. Participation of ROS in Varicose Veins

Activated leukocytes, mainly neutrophils, adhered to the endothelial surface to release ROS [6,7]. These cells release superoxide, the dismutation of which leads to the production of hydrogen peroxide, hydroxyl radicals, nitric oxide, and other nitrogen compounds (nitrogen dioxide, peroxynitite); singlet oxygen and hypochlorous acid are also produced. HClO or myeloperoxidase activity is a frequently used indicator of neutrophil activation [150,151]. In varicose veins, myeloperoxidase activity was significantly higher than in normal veins [1]. Activated neutrophils and monocytes, in addition to releasing ROS, also produce large amounts of proteases and phospholipases, which are also damaging factors to the vessel wall and the surrounding tissues. Furthermore, the interaction of activated neutrophils with the endothelium results in the conversion of xanthine dehydrogenase to xanthine oxidase in endothelial cells [152]. XO oxidizes hypoxanthine to xanthine, generating superoxide, and then xanthine to uric acid, generating another O_2_ molecule [153]. ECs also have XO and can produce ROS [154]. ROS are also produced by ECs via NADPH oxidase and nitric oxide synthase [155,156]. An increase in XO expression was observed in the walls of varicose veins with superficial thrombophlebitis [7]. A higher level of TBARSs, which is an indicator of lipid peroxidation, was found in the varicose vein wall and in the blood [6]. In addition, a decrease in antioxidant potential and a decrease in SOD activity were demonstrated in damaged veins [6]. A decrease in antioxidant potential in plasma and SOD activity was also observed in erythrocytes from varicose veins and in the varicose vein wall. On the other hand, an increase in SOD activity was observed in the varicose vein wall [157]. Increased NO levels were observed in more severe VV conditions (lipodermatosclerosis and healed ulceration) [158]. A decrease in non-enzymatic antioxidant capacity (NEAC) was also demonstrated in the plasma collected from varicose veins compared that collected from peripheral veins. Varicose vein plasma was characterized by a lower level of thiols. A decrease in -SH groups was also observed in the hemolysate and erythrocyte plasma membranes. At the same time, an increase in plasma and RBC carbonyl compounds and TBARS levels was recorded, as well as a decrease in catalase and acetylcholinesterase (AChE) activity. A decrease in AChE activity was also observed in the aging process and inflammation [159]. These results show that ROS-induced damage occurs not only in varicose blood but also in red blood cells [8].

ROS also leads to damage to subendothelial tissue, hyperplasia of smooth muscle cells, and causes increased endothelial permeability [160]. Reactive oxygen species are generated in hypoxia during purine catabolism when ATP is transformed into hypoxanthine and xanthine. Hypoxia and inflammation lead to the expression of cytokines (TNF-α, IFN-γ, IL-6, and IL-1) and the activation of xanthine oxidoreductase (XOR) gene transcription, which is involved in purine catabolism [161,162]. XOR is a multi-level regulated enzyme and its mechanism of action in the production of ROS is similar to xanthine oxidase [153,163]. Moreover, it has nitrite reductase activity, generating nitric oxide, causing vasodilation and regulation of blood pressure. On the other hand, adhesion and activation of neutrophils and monocytes leads to an oxygen burst generating a variety of reactive oxygen species [99].

### 4.7. Venous Leg Ulcer

VLU is a consequence of CVD caused by reflux, blood stasis, venous hypertension, and inflammation that results in dermatological complications. Over time, they can lead to complications such as infections and delayed wound healing. In addition, varicose veins lead to impaired macro- and microcirculation [164]. However, despite the fact that chronic venous disease occurs in about 1/3 of the population, ulcers are relatively rare and occur in only about 5% of cases, but their causes are not fully understood [165]. The problem is non-healing ulcers, in which increased expression of many proteins is observed, such as frizzled-related protein 4, branched-chain aminotransferase 1, dermatopontin, cytochrome P450, and 17 B hydroxysteroid dehydrogenase, which is involved in the control of inflammation, signaling, the growth of cells, the assembly of the extracellular matrix, and steroidogenesis [166,167].

In patients with varicose veins and skin lesions in CVD, as well as with deep vein insufficiency, a significantly increased risk of ulceration is observed. Additionally, the risk may also be increased in people who smoke, are obese, have limited ankle mobility, or have limited calf muscle pump power [168]. In leg ulcers, the expression of genes mainly related to the inflammatory stage has been identified. These genes have not been previously associated with wound healing. Biopsies from non-healing venous ulcers revealed deregulation of keratinocyte activation and differentiation pathways in the epidermis [167].

It is believed that an imbalance in redox homeostasis in chronic wounds leads to a non-healing state. ROS are released mainly by the activated neutrophils and macrophages and to a lesser extent by venous fibroblasts and endothelial cells. Inflammation in chronic wounds, as well as ROS levels, persist for a long time, and the consequence is permanent damage to the host’s biological material and perpetuation of inflammation [169]. Oxidative stress is considered to be one of the important pathophysiological factors that occur during impaired wound healing in venous stasis ulcers. Significantly reduced capacitance was observed in the plasma of patients with antioxidants (AOs) in comparison with healthy individuals. A significant difference in AO values was also demonstrated in patients with diabetes (DM) and chronic venous ulcers compared to patients without (DM) and chronic venous ulcers [170]. In samples taken from venous ulcers, a significant increase in MDA as a marker of oxidative stress was found. Interestingly, no differences were found in the GSH level and GSH/GSSG ratio. However, the presence of Fe in the form of hemosiderin was found [171].

## 5. Role of Red Blood Cells in Varicose Veins

Due to their function, which is related to the transport of oxygen to cells and tissues, RBCs are exposed to both external and internal sources of reactive oxygen species. ROS lead to changes in structure and function, affecting RBC integrity and impairing oxygen delivery to cells and tissues. The properties of red blood cells and the oxidative and antioxidant systems in these cells have recently been extensively described by Möller et al. [172]. There are several red blood cell defects and situations that generate states of oxidative stress in which the defense mechanisms are overloaded. These include glucose-6-phosphate dehydrogenase deficiencies (favism), hemoglobinopathies such as sickle cell disease and thalassemia, and packed red blood cells for transfusion that undergo storage changes. RBCs participate in thiol metabolism through the capture of thiols and transport of dithiols. RBCs express 3-mercaptosulfotransferase, which is associated with the endogenous production of thiols and their metabolites [173].

**Figure 3:**
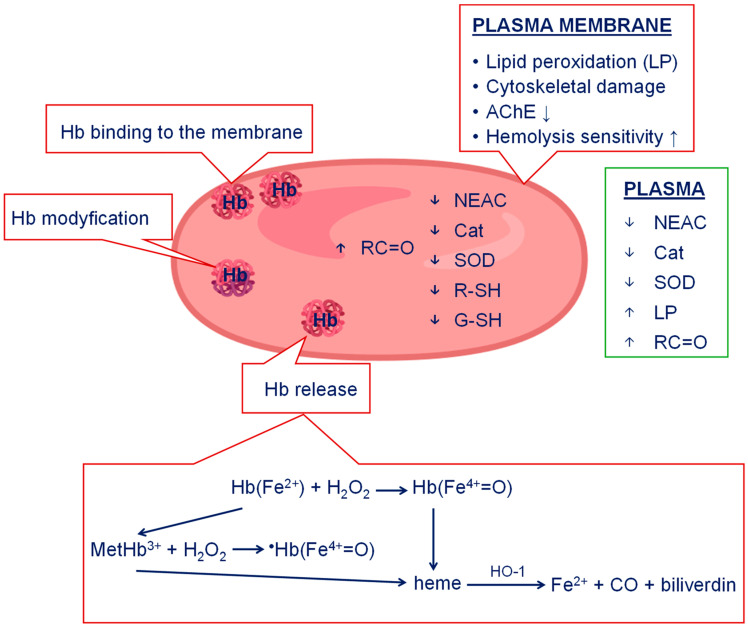
Red blood cells in varicose veins in hypoxic conditions of blood stasis. Reduced activity of antioxidant enzymes (SOD, CAT), decreased levels of low molecular weight antioxidants such as glutathione (GSH), ascorbate (ASC), α-tocopherol (α-TOH), and thiols, and decreased non-enzymatic antioxidant capacity (NEAC). The potential is lower than the levels of GSH, ASC, TOH, and thiols. Damaged hemoglobin (Hb) as a result of oxidative stress inside RBCs. Binding of damaged hemoglobin to the cytoplasmic part of the cell membrane. In the oxidatively damaged RBC plasma membrane, an increase in lipid peroxidation (LP), an increase in the level of carbonyl compounds (RC=O), a damaged membrane cytoskeleton, a decrease in acetylcholinesterase (AChE) activity, and an increase in RBCs sensitivity to hemolysis was found. Released hemoglobin from the red blood cell is sensitive to oxidation to methemoglobin (MetHb). On the other hand, hydrogen peroxide oxidizes Hb and MetHb to the ferryl form and the free radical ferryl form, respectively. MetHb and the ferryl form of hemoglobin release heme, which decomposes under the influence of heme oxidase (HO-1) with the release of free Fe^2+^ ions, a catalyst for the Fenton reaction and other free radical reactions. In plasma collected from varicose veins, reduced activity of antioxidant enzymes (SOD, Cat), decreased NEAC, lipid peroxidation, and carbonyl compound levels were observed.

Already at the end of the 19th century, Biernacki had demonstrated faster sedimentation in an inflammatory state compared to the normal state [174]. This test, combined with a clinical interview and examination of the patient, can be helpful not only in diagnosis but also in treatment and monitoring the course of autoimmune diseases, as well as acute and chronic infections and even neoplastic diseases [175]. Today, it is known that the red blood cells of healthy people are responsible for stimulating the activity and maturation of immune cells. On the other hand, RBCs from sick people are devoid of this function. It is known that RBCs bind over 50 cytokines and serve as their absorber. A decrease in cytokine binding is associated with the progression of the disease. RBCs also have a receptor on their surfaces that binds chemokines. Eleven chemokines associated with RBCs have been identified to date. RBCs also bind cholesterol and other lipids [176]. Although RBCs participate in many pathologies, the mechanism of their participation has not yet been explained. It has been reported that red blood cells have an immunostimulatory effect on some cell populations [177]. It has been shown that after transfusions they can cause a cytokine storm [177,178]. Studies conducted on whole erythrocytes collected from varicose veins showed a decrease in the internal viscosity of RBCs. A decrease in the fluidity of RBCs plasma membrane lipids was also demonstrated. In addition, disturbances in the conformational state of the red blood cell membrane cytoskeleton were found (Figure 3). The observed changes were correlated with changes in RBC osmotic resistance. Erythrocytes from varicose veins showed greater sensitivity to hemolysis compared to erythrocytes collected from the antecubital vein of the same patients [179]. In varicose veins, an overload of the damaged vein with iron occurs. A higher serum iron concentration was found in patients with ulcers compared to patients in CEAP stage C4, which may be related to their loss of mechanisms preventing the release of iron from phagocytes [6].

### 5.1. Hemoglobin and Myoglobin as ROS Catalyst

The autoxidation of oxyhemoglobin is the major source of endogenous RBC oxidant production, leading to the generation of superoxide radical and then hydrogen peroxide. In addition, strong oxidants from other blood cells and the surrounding endothelium can reach RBCs. Abundant and efficient enzyme systems and low molecular weight antioxidants prevent most of the damage to RBCs and also position RBCs as a scavenger of vascular oxidants that allow the body to maintain a healthy circulatory system. Among the antioxidant enzymes, the thiol-dependent peroxidase peroxiredoxin-2, which is abundant in RBCs, is essential for maintaining the redox balance. Much of the antioxidant activity of RBCs is supported by active glucose metabolism, which provides reducing power in the form of NADPH via the pentose phosphate pathway. These oxidative-stress-related RBC pathologies underscore the importance of redox balance in these anucleated cells, which lack a DNA-induced antioxidant response mechanism and rely on a complex and robust network of antioxidant systems. In many disease states, red blood cell hemolysis is observed [145]. Extravasated hemoglobin is sensitive to oxidation.

Myoglobin (Mb) is a heme protein that transports oxygen in skeletal muscle and the heart of vertebrates. In addition, myoglobin, which has a greater affinity for oxygen, is its storage, compensating for the lack of oxygen associated with the reduced blood flow through the heart and skeletal muscle during contraction [180].

Red blood cells are also exposed to oxidants from the external environment that are produced by endothelial cells and the immune system. Vascular endothelium releases O_2_^•−^, ^•^NO, ONOO^−^, H_2_O_2_, and HOCl. The two oxygen-carrying proteins (respiratory proteins) found in red blood cells and muscle tissues, i.e., Hb and Mb, are maintained in a reduced state by numerous systems such as hemoglobin reductase and metmyoglobin reductase. Despite the presence of the enzyme reducing 3% of Hb daily, it undergoes autoxidation to methemoglobin (MetHb) [181].

$$Hb({Fe}^{2+}O_{2})\to Hb({Fe}^{3+})+O_{2}^{•-}$$

However, in pathological conditions, hemoglobin and myoglobin are released from cells, which causes separation from cellular reduction systems and consequently leads to their oxidation. In addition to the formation of met forms (MetHb and MetMb), in the case of oxidants such as peroxides, ferryl forms, and radical ferryl forms can be formed in which the iron ion occurs in the fourth oxidation state [182].

$$Hb/Mb({Fe}^{2+}O_{2})\to Hb/Mb({Fe}^{3+})+O_{2}^{•-}$$

Superoxide anion radicals form hydrogen peroxide by spontaneous or catalyzed dismutation (SOD):

$${2H}^{+}+2O_{2}^{•-}\to H_{2}O_{2}$$

Inorganic and organic peroxides oxidize Hb and MetHb to iron forms in higher oxidation states. Ferryl radical forms of Hb are a mixture of radicals with an unpaired electron on globin and porphyrin, as well as cross-linked forms of Hb. In addition, oxidized forms of Hb release heme, which is also a pro-oxidant factor. Hydrogen peroxide oxidizes myoglobin and hemoglobin, respectively, to oxoferryl forms in which iron occurs in the fourth oxidation state [182].

$$Hb/Mb({Fe}^{2+})+H_{2}O_{2}\to Hb/Mb({Fe}^{4+}=O^{2-})$$

In turn, MetMb and MetHb are oxidized to a cation radical which quickly deprotonates to form the radical (Fe^4+^=O_2_^−^)^•^R [183].

$$Hb/Mb({Fe}^{3+})+H_{2}O_{2}\to Hb/Mb{\left({Fe}^{4+}=O^{2-}\right)}^{•+}$$

Such a form was found in venous blood collected from healthy adults using EPR spectroscopy [184]. On the other hand, oxoferryl forms have peroxidase properties, catalyzing the decomposition of hydrogen peroxide:

$$Hb/Mb({Fe}^{4+}=O^{2-})+H_{2}O_{2}\to Hb/Mb({Fe}^{3+})+O_{2}^{•-}$$

However, oxyferryl and free radical forms occur in acute renal dysfunction (also referred to as acute (renal failure)) after rhabdomyolysis. Rhabdomyolysis is a condition that occurs as a result of mechanical muscle damage (crushing) or chemical lysis of muscle cells, releasing Mb [185]. The myoglobin released from myocytes, deprived of the protective conditions prevailing inside the cells, is oxidized by exogenous oxidants to form iron in the fourth oxidation state.

Iron compounds in a higher oxidation state are reactive intermediates and are easily decomposed [186]. The ferryl form of hemoglobin can lead to the formation of globin radicals due to intramolecular electron transfer between the Fe^4+^=O ion and some amino acids in the globin chains. Globin radical reactions lead to cross-linking of oxidized hemoglobin derivatives [187].

$$Hb({Fe}^{4+}=O)+{2H}^{+}\to {\left(Hb{Fe}^{3+}\right)}^{•+}+H_{2}O_{2}\;{\left(Hb{Fe}^{3+}\right)}^{•+}+{\left(Hb{Fe}^{3+}\right)}^{•+}\to \left(Hb{Fe}^{3+}\right)\text{-}{\left(Hb{Fe}^{3+}\right)}^{+}$$

The ferrylHb forms are a strong pro-inflammatory factor that has a major effect on vascular endothelial cells. However, extravasated hemoglobin is bound by haptoglobin (Hp) and removed from the circulation by endocytosis into macrophages via the CD163 receptor [188,189]. The Hb-Hp complex inhibits the release of heme from MetHb and is less sensitive to hydrogen peroxide. However, if hemoglobin is oxidatively modified (ferrylHb), then its removal by Hp is severely impaired.

The release of heme occurs as a result of hemoglobin oxidation, heme is strongly toxic to cells. Damage to endothelial cells by heme was demonstrated, involving polymorphonuclear leukocytes and ROS. Therefore, Hb present in plasma is the main source of heme, increasing the sensitivity of cells to oxidative damage. One line of defense against heme and Hb is the expression of heme oxygenase-1 and ferritin by endothelial cells. Heme oxygenase-1 (HO-1) degrades heme, and ferritin binds the released iron ion [190]. Heme can also be bound by hemopexin (Hx), an acute phase plasma protein, which inhibits the prooxidant activity of heme [191,192]. In turn, the Hx-heme complex is taken up by the LDL scavenger receptor CD91 of hepatocytes and macrophages in the liver and spleen [193].

### 5.2. Binding of Nitric Oxide by Hemoglobin

The released nitric oxide in the blood is bound by heme in hemoglobin due to its high affinity for iron. While deoxyhemoglobin binds NO without changing the oxidation state of iron, Hb is oxidized to methemoglobin. The reaction proceeds to form the peroxynitrito-complex HbFe^3+^OONO, which rapidly decomposes into MetHb and nitrate ion:

Hb+NO•→HbNOHbFe2+O2+NO•→HbFe3+OONO → HbFe3++NO3−

The reaction rate constant is high and equal to k = 8.93 × 10^7^ M^−1^ s^−1^. NO^•^ also binds to methemoglobin with a rate constant k = 1.71 × 10^3^ M^−1^ s^−1^ to form the HbFeII(NO^+^) complex [194].

$$\mathrm{Hb}{Fe}^{3+}+NO\to \mathrm{Hb}{Fe}^{3+}NO\to \mathrm{Hb}{Fe}^{2+}{\left(NO\right)}^{+}$$

Nitric oxide also reacts with the ferryl form of hemoglobin, which is rapidly reduced by NO^•^ to MetHb and nitrite (rate constant k = 2.4 ± 0.2 × 10^7^ M^−1^ s^−1^) [195].

$$Hb\left({Fe}^{4+}=O\right)+NO\to Hb{Fe}^{3+}ONO\to Hb{Fe}^{3+}+{NO}_{2}^{-}$$

However, ^•^NO bioactivity in RBCs is maintained because it is also bound by a cysteine Cysβ93 residue of oxyHb present in the globin chain leading to the formation of S-nitrosohemoglobin (HbSNO) [195,196]. Red blood cells participate in vasodilation, which is related to the autoregulation of blood flow and is related to the dilation of blood vessels in hypoxia, which has to increase oxygen delivery. This mechanism is related to the binding of nitric oxide by hemoglobin to form nitrosohemoglobin. HbSNO formation is of particular importance in the regulation of local blood flow because ^•^NO release from Hb is synchronized with the release of oxygen [197]. In addition to transporting oxygen, the formed HbSNO also enables the dilation of blood vessels. During the deoxygenation of hemoglobin, ^•^NO is released from HbSNO, which allows for regulation of blood flow in vessels [198].

## 6. Summary

In this work, we showed the involvement of oxidative stress in the pathophysiology of varicose veins and the participation of red blood cells in this process. In this disease, ROS is produced from many sources. We showed that hypoxia and inflammation contribute to the increased production of ROS. Large amounts of ROS are produced directly by monocytes and neutrophils during the oxygen burst. In addition, we are dealing with the expression of ROS-producing enzymes such as NADH oxidase, xanthine oxidase, oxidoreductase, myeloperoxidase, and iNOS in the endothelium and other cells. We have also shown the participation of red blood cells in the pathology of VVs and revealed the pathological participation of hemoglobin released from RBCs in the overproduction of ROS. In addition to numerous sources of oxygen, in this pathology, there is also damage to the entire antioxidant system. This consists of a decrease in the activity of enzymes such as SOD, Cat, GPx, and others. Also the decrease in the concentration of low molecular weight antioxidants such as glutathione, thiols, ascorbic acid, tocopherols, carotenoids, and others. The ROS production and removal imbalance leads to oxidative damage to biological material, as indicated by elevated levels of TBARS and carbonyl compounds. In addition, varicose veins express proteolytic enzymes such as proteases and metalloproteinases, which also contribute to the damage of important molecules and macromolecules.

## Figures and Tables

**Table 1:** Enzymatic and low molecular weight antioxidants.

Enzyme	Role	Low Molecular Weight Antioxidants	Role
superoxide dismutase	superoxide anion radical scavenger	glutathione	free radical scavenger, removes ONOO^−^ and HClO, substrate of many enzymes, maintains redox state in cells, regulator of homeostasis
catalase	hydrogen peroxide scavenger	ascorbic acid	free radical scavenger, regenerates tocopheroxyl radical
glutathione peroxidase	hydrogen peroxide and organic hydroperoxide scavenger, simultaneous oxidation of glutathione	α-tocopherol	lipid radical scavenger
glutathione reductase	reduction of oxidized glutathione	β-carotene	lipid radical scavenger, α-TOH regenerates β-carotene radical
peroxiredoxin	reduction of hydrogen peroxide and organic hydroperoxide	lipoic acid	free radical scavenger, removes ONOO^−^ and NO_2_ HClO scavenger
thioredoxin reductase	reduction of oxidized peroxiredoxin and -S-S- bridges in proteins	dihydrolipoic acid	free radical scavenger, removes ONOO^−^ and NO_2_
glutaredoxin (thioltransferase)	reduction of peroxiredoxin and dehydroascorbate	uric acid	free radical scavenger, removes ONOO^−^ and HClO

## Abbreviations

α-TOH	α-tocopherol
AChE	acetolocholinesterase
AO	antioxidant
Asc	ascorbic acid
CAMs	cell adhesion molecules
Cat	catalase
CVD	chronic cardiovascular disease
CVI	chronic venous insufficiency
DHLA	dihydrolipoic acid
DM	diabetes mellitu
DVT	deep vein thrombosis
eNOS	endothelial NOS synthase
EPO	eosinophil peroxidase
ET-1	aldosterone, endothelin-1
ETC	electron transport chain
GPx	glutathione peroxidase
Grx	glutaredoxin
GSH	reduced form of glutathione
GSSG	oxidized form of glutathione
Hp	haptoglobin
Hb	hemoglobin
HbSNO	S-nitrosohemoglobin
HIF	hypoxia-inducible factors
HO-1	heme oxygenase-1
HUVEC	human umbilical vein endothelial cells
HVVEC	human varicose vein endothelial cells HVVEC
Hx	hemopexin
ICAM-1	intracellular adhesion molecule-1
ICAM-2	intracellular adhesion molecule-2
JAM	junctional adhesion molecule
LA	lipoic acid
LMWA	low molecular weight antioxidants
LTB4	leukotriene B4
Mb	myoglobin
MDA	malondialdehyde
MetHb	methemoglobin
MetMb	metmyoglobin
MMP	matrix metalloproteinase
MPO	myeloperoxidase
NEAC	non-enzymatic antioxidant capacity
NETs	extracellular neutrophil traps
NOS	nitric oxide synthases
NOX	NADPH oxidase
OPPC	oxidative pentose phosphate cycle
PDGF	growth factor
PDT	photodynamic therapy
PE	pulmonary embolism
PMNs	polymorphonuclear leukocytes
Prx	peroxiredoxin
PUFA	polyunsaturated fatty acids
RBCs	red blood cells
ROS	reactive oxygen species
SMC	smooth muscle cells
SOD	superoxide dismutase
TBARS	thiobarbituric acid-reactive substances
TGFβ	transforming growth factor β
TNFα	tumor necrosis factor α
VLU	venous leg ulcers
VTE	venous thromboembolism
VV	varicose vein
XO	xanthine oxidase
XOR	xanthine oxidoreductase
